# Biliptysis Revealing a Bilio-Bronchial Fistula

**DOI:** 10.7759/cureus.42842

**Published:** 2023-08-02

**Authors:** Afaf Thouil, Tariq Bouhout, Meriem Rhazari, Badr Serji, Hatim Kouismi

**Affiliations:** 1 Department of Respiratory Diseases, Research and Medical Sciences Laboratory, Faculty of Medicine and Pharmacy of Oujda, Mohammed VI University Hospital, Mohammed First University, Oujda, MAR; 2 Department of Surgical Oncology, Mohammed VI University Hospital, Regional Oncology Center, Oujda, MAR

**Keywords:** endoscopic sphincterotomy, pericystectomy, hepatic hydatidosis, bilio-bronchial fistula, biliptysis

## Abstract

The presence of a bilio-bronchial fistula (BBF) of hydatid origin is considered a serious complication as it can lead to significant injuries at the abdominal, diaphragmatic, and thoracic levels. Here, we report the case of a 70-year-old patient presenting with biliptysis as a symptom and whose thoracic and abdominal CT scan confirmed the presence of a right BBF. The management consisted of an initial endoscopic sphincterotomy, followed by an exclusive left thoracotomy surgery to treat lung, liver, and diaphragmatic injuries. Fortunately, the evolution was favorable with the disappearance of the biliptysis. To diagnose a BBF, it is crucial to conduct a precise assessment, focusing mainly on imaging to accurately locate the injury before any surgical intervention.

## Introduction

A bilio-bronchial fistula (BBF) refers to an abnormal communication between the biliary tract and the tracheobronchial tree. Although it can occur in other hepatobiliary disorders, this condition is often associated with hepatic hydatidosis. It is a dreaded complication of hydatid cysts of the liver that rupture into the thorax, and it can cause serious injuries at the abdominal, diaphragmatic, and thoracic levels, with high perioperative mortality. The presence of bilioptysis is pathognomonic for a bronchobiliary fistula [1,2]. The resulting broncho-pulmonary and hepatobiliary injuries are particularly concerning [1]. Our main goal is to emphasize the significance of early detection and implementation of suitable treatment strategies in order to minimize surgical mortality rates and enhance overall patient well-being.

## Case presentation

We report the case of a 72-year-old patient being treated for high blood pressure and had undergone a cholecystectomy one month prior. She was admitted for the management of exertional dyspnea with productive cough producing greenish expectoration, all occurring in a context of unquantified fever. Upon admission, the clinical examination found a conscious patient, with resting polypnea at 28 cycles/min, and her saturation was measured at 94% in ambient air, tachycardia at 105 bpm, blood pressure = 100/50 mm Hg, afebrile condition, and generalized cutaneomucosal pallor with generalized cutaneous jaundice.

**Figure 1:**
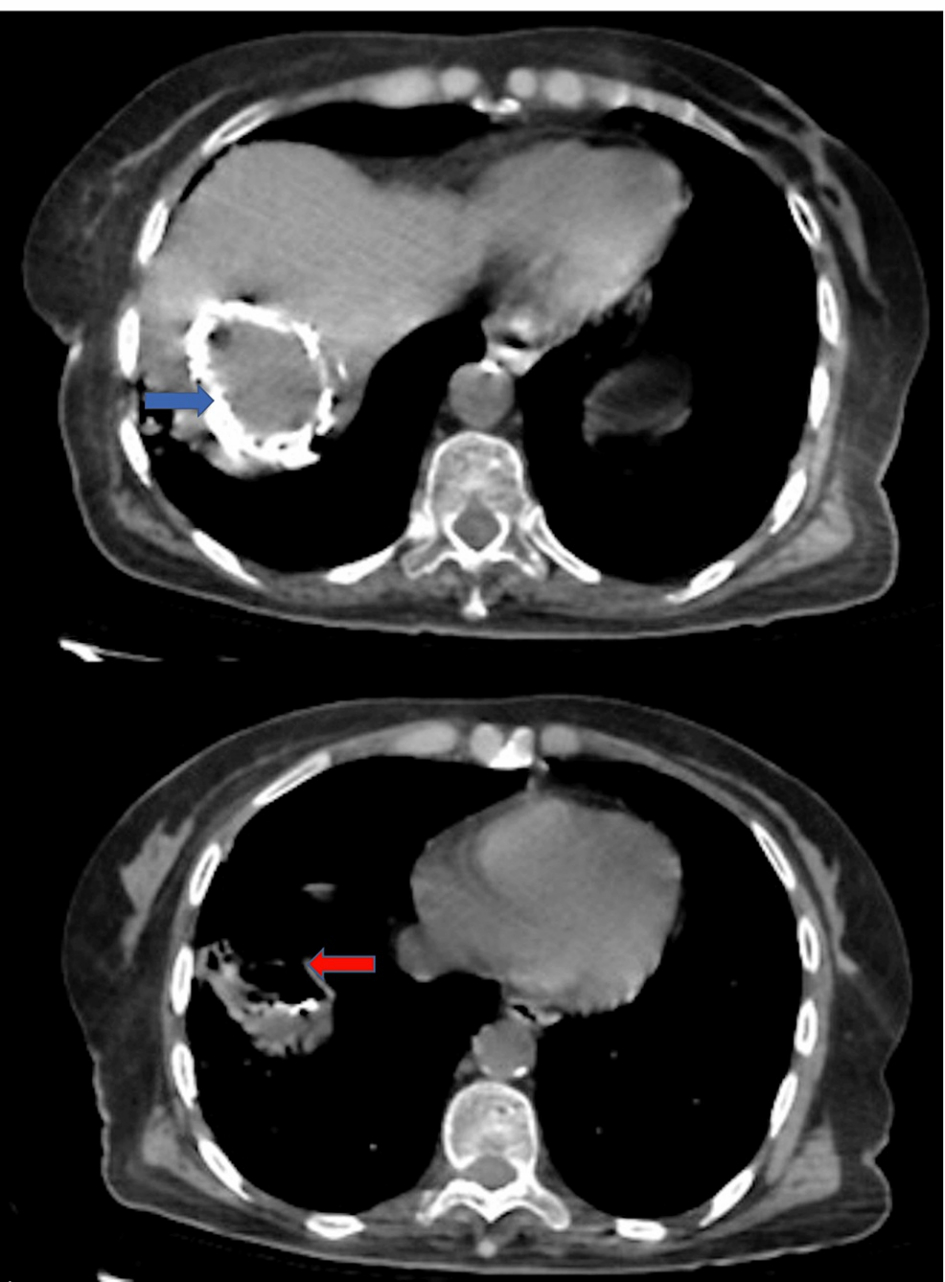
Thoraco-abdominal CT scan with and without contrast showing a bilio-bronchial fistula (red arrow) associated with a hepatic cystic lesion of segment VII (blue arrow).

The pleuropulmonary examination revealed bilateral basal crepitant and snoring rales, and the rest of the examination results were normal. The thoraco-abdominal CT scan with and without contrast showed a BBF with multiple pulmonary nodules and micronodules associated with a hepatic cystic lesion of segment VII (Figure 1).

**Figure 2:**
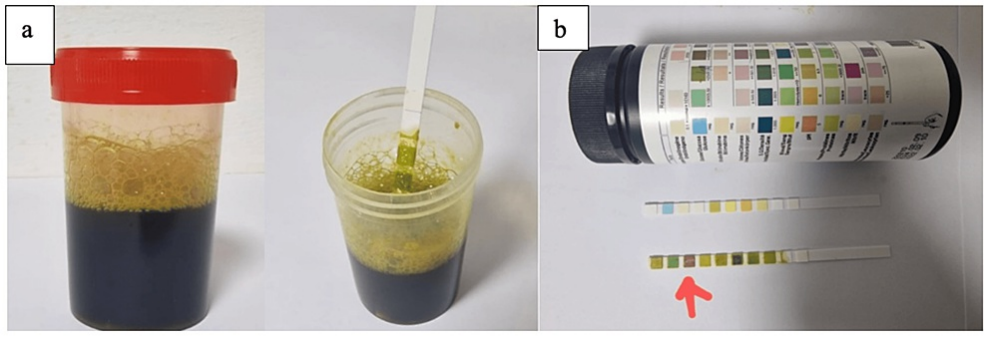
Specimen images showing biliptysis (a). The Labstix test in the bronchial secretions was highly positive for bilirubin (b).

Biologically, the patient had anemia with a hemoglobin level of 8.6 g/dl and C-reactive protein (CRP) = 134 with a normal liver panel, and hydatid serology was positive. The Labstix test in the bronchial secretions was highly positive for bilirubin (Figure 2).

Given this situation, an opinion from a visceral surgeon was sought, and the management consisted of a transabdominal approach. Intraoperatively, we found a large cyst occupying segment VII with a small breach in the right diaphragmatic dome. We performed a pericystectomy and occlusion of a diaphragmatic breach, the operative time was 120 min, and the blood loss was 100 cc. Afterward, the patient underwent an endoscopic sphincterotomy. Pathology of the resected specimen showed a cystic wall with a mononuclear inflammatory infiltrate mononuclear infiltrate of lymphoplasmocytes in contact with hydatid membranes. The patient did not receive albendazole after the surgical procedure. One month later, the patient's health significantly improved, with the disappearance of biliptysis symptoms, resolution of respiratory symptoms, and good radiological improvement.

## Discussion

A BBF is a rare disease, with a frequency ranging from 2.5% to 16% according to authors, and it is known to be caused by different causes: congenital, malignancies, abscesses, traumatic, or iatrogenic [2]. The presence of biliptysis is a specific sign of bronchobiliary fistula, which can be found in 12.5% to 77.8% of patients according to case series [3,4,5]. Other signs, such as vomica, may also indicate a rupture of a hepatic hydatid cyst into the lung. Digestive signs, such as jaundice, are not specific and do not allow for an accurate diagnosis. In the case of biliptysis, thoracic and abdominal CT scan is the examination of choice to assess lesions at the liver, lung, and diaphragm levels, as well as the resulting damage. Bronchoscopy can be useful to evaluate the severity of bronchial tree lesions and determine the origin of biliptysis [6]. In this case, CT scan accurately located the origin of the biliptysis and therefore that of the BBF.

A definitive treatment for BBFs has not yet been established. Surgical or non-surgical interventional procedures, such as endoscopic retrograde cholangiopancreatography (ERCP) or percutaneous transhepatic drainage (PTD), are frequently used as direct photographic evidence and management [7]. Transhepatic embolization, bronchoscopic injection of n-butyl cyanoacrylate, and histoacryl embolization have also been performed [8]. The treatment of hydatid-origin BBFs aims to dry up the fistula and treat the underlying cause, and it requires adequately prepared surgical intervention [6].

Thus, suitable antibiotic therapy and good respiratory physiotherapy are necessary to fight infections, and blood transfusion and hydroelectrolytic and caloric rebalancing may also be necessary. The surgical treatment has five main objectives: to treat intrathoracic lesions, to treat liver lesions after hepato-diaphragmatic disconnection, to search and treat biliary fistulas, to repair the diaphragm, and to adequately drain the pleural and hepatic cavity. Several approaches have been proposed for this surgical intervention, such as thoracotomy alone, thoraco-phreno-laparotomy, laparotomy alone, or laparotomy associated with a thoracotomy. Although the abdominal route is often sufficient to control the entirety of the lesions, as was the case with our patient, some authors recommend thoracotomy for significant pleuropulmonary lesions requiring pulmonary resection [6].

Due to the septic nature of BBF surgery, it is understandable that the incidence of postoperative infectious complications is high. The postoperative morbidity and mortality rate remains high, ranging between 12.2% and 50% according to studies [6,9].

## Conclusions

A BBF is a rare condition characterized by communication between the biliary duct and the bronchial tree, causing bilioptysis. It can be congenital or secondary to trauma, biliary duct obstruction, or hepatic diseases, such as tumor invasion. Recurrent pulmonary infections can occur. BFF is a challenging medical condition that requires an awareness of its existence and possible etiologies to improve its evaluation and treatment. There are several treatment options, from conservative drainage to more invasive interventions through bronchofibroscopy or cholangiography, with variable results.
